# Myocarditis Associated with COVID-19 Vaccination

**DOI:** 10.3390/vaccines12101193

**Published:** 2024-10-19

**Authors:** Kamila Florek, Mateusz Sokolski

**Affiliations:** 1 Student Scientific Club of Transplantology and Advanced Therapies of Heart Failure, Institute of Heart Diseases, Faculty of Medicine, Wroclaw Medical University, 50-369 Wroclaw, Poland; 2 Institute of Heart Diseases, Faculty of Medicine, Wroclaw Medical University, 50-556 Wroclaw, Poland; 3 Centre for Heart Diseases, University Hospital, 50-556 Wroclaw, Poland

**Keywords:** myocarditis, COVID-19, COVID-19 vaccines, 2019-nCoV vaccine mRNA-1273, mRNA vaccines

## Abstract

Myocarditis after the COVID-19 vaccine is one of the important adverse events following immunization, observed mainly after mRNA-based vaccines. Importantly, post-vaccination myocarditis was less common than myocarditis after SARS-CoV-2 infection, as it was scored at 19.7 per 1,000,000 doses and 2.76 per 1000 infections. Predominantly, its course was benign and, compared with the myocarditis after COVID-19 infection, significantly fewer patients developed heart failure or died among patients with post-vaccination myocarditis. The group at highest risk of myocarditis related to COVID-19 vaccination were young males who received a second dose of an mRNA vaccine. It was observed that, among mRNA vaccines, specifically mRNA-1273 was associated with a higher risk of myocarditis. The mechanism underlying myocarditis after COVID-19 vaccination is still under investigation and certain processes are being considered. Currently, some follow-up assessments of patients who developed vaccine-induced myocarditis are available and suggest a favorable prognosis. The aim of this review is to discuss the most recent data on myocarditis after COVID-19 vaccination considering its epidemiology, clinical presentation, diagnosis, management, relative risk of myocarditis compared with SARS-CoV-2 infection, potential underlying mechanism, and follow-up data of patients who developed post-vaccination myocarditis.

## 1. Introduction

Acute myocarditis is commonly defined as a sudden inflammatory injury to the myocardium [1]. The leading cause of myocarditis is viral infection, followed by systemic inflammatory diseases, then medications—mainly antipsychotics and immunotherapeutics—and finally, it can be caused by vaccines [1,2]. Myocarditis presentation varies from mild symptoms resolving in 50% without treatment, to life-threatening arrhythmias, cardiogenic shock, and acute heart failure (HF), with 25% of cases requiring a ventricular support approach or heart transplantation [3,4].

Vaccine-related myocarditis before the COVID-19 vaccines era was described mainly according to immunization against smallpox and influenza, as well as in several cases of pneumococcal, tetanus, cholera, and rabies vaccinations [5,6,7]. However, a peak in the data about post-vaccination myocarditis in the PubMed database timeline was related to COVID-19 vaccines. There was a significant association between vaccine type and occurrence of myocarditis highlighting the dominance of mRNA-based ones [8]. Although intensive, population COVID-19 vaccinations were organized in a relatively short period, due to the emerging pandemic, and their side effects might accumulate in time compared to non-COVID vaccines. Hence, it was shown that the occurrence of myocarditis following COVID-19 vaccinations was not significantly higher than in the non-COVID-19 vaccine receivers [9]. Furthermore, the risk of myocarditis was significantly greater after smallpox vaccinations and did not differ for other vaccines compared to COVID-19 vaccines [9]. According to the most recent data, conventional myocarditis incidence was assessed as 1–10 cases per 100,000 individuals, after SARS-CoV-2 infection it was scored as 2.76 per 1000, while myocarditis related to mRNA vaccination was recently estimated as 19.7 per 1,000,000 doses [10,11]. Even though some adverse effects of COVID-19 vaccines have been noted, they were rarely severe [12]. Regarding myocarditis after vaccination against SARS-CoV-2, it had a mainly benign course and favorable prognosis [13,14]. Importantly, at the population level, these vaccines reduced the risk of death due to SARS-CoV-2 infection without increasing the risk of death from other causes in each age group [15,16]. The World Health Organization (WHO) has estimated that vaccines reduced COVID-19 mortality by 57% and saved 1.4 million lives in the European region [17].

This study aimed to provide a comprehensive review of the most recent data about myocarditis after COVID-19 vaccination considering its epidemiology, clinical presentation, diagnosis, management, the relative risk of myocarditis compared with SARCoV-2 infection, potential underlying mechanism, and follow-up data of patients who developed post-vaccination myocarditis.

## 2. Methods

The review was conducted by searching PubMed considering all of the articles available from 2019 with keywords: myocarditis, COVID-19, COVID-19 vaccines, 2019-nCoV vaccine mRNA-1273, mRNA vaccines, immunization, post-vaccination myocarditis. The inclusion criteria were (I) Studies related to myocarditis after COVID-19 vaccination; (II) English language; (III) publication in peer-reviewed journals; (IV) human population. We excluded animal studies and abstracts without complete text publications or single articles that were already involved in the meta-analysis considered in this review. The most relevant meta-analysis, randomized controlled trials, cohort studies, and case reports published between April 2020 and May 2024 were assessed by an independent reviewer. The articles were evaluated according to the title, abstract, text, and scientific validity.

## 3. Available COVID-19 Vaccines and the Differences in the Risk of Myocarditis

The emerging COVID-19 pandemic was the reason why vaccines against the virus were approved in an accelerated process. The standard vaccine acceptance timeline spans 5–10 years, whereas regarding the first COVID-19 vaccine, the procedure was completed in under a year without skipping any steps [18,19]. The vaccine development process consists of the following phases: preclinical trials performed mainly in animal models, phase I-III clinical trials which can be combined under pandemic circumstances, and the regulatory approval process which is completed if the benefits from vaccination are greater than possible risks, followed by large scale production and studies after authorization [18]. Phase I clinical trials assess safety, dosing, and immune responses. In the accelerated evaluation this phase can be completed within 2–3 months, compared to 1–10 years in the standard protocol [18]. Phase II clinical trials also assess safety and immune effectiveness (completed within 2–3 years in the standard, and 3–4 months in an accelerated pathway), focusing on the most common side effects, but in a larger group and a more diverse population, importantly according to age criteria starting with the adult population moving progressively toward younger groups [18,20]. Phase III clinical trials also serve to understand vaccine safety and effectiveness (completed within 2–4 years in a standard and 6–9 months in an accelerated pathway), including long-term effects observed over at least two years of follow-up, with participants at this stage randomized to the vaccine and placebo groups [18].

There are different types of COVID-19 vaccines including mRNA, viral vector, and protein-based vaccines, listed in order of their approval [21]. Currently, six COVID-19 vaccines are authorized by the European Medicines Agency (EMA) for use in the European Union (EU) [22]. Myocarditis related to COVID-19 vaccination was noticed in the majority after mRNA vaccines BNT162b2 (Corminaty, Pfizer) and mRNA-1273 (Spikevax, Moderna), which constitute 80% of all COVID-19 vaccine doses administered in the EU [23]. These vaccines consist of mRNA encoding for the spike glycoprotein of SARS-CoV-2, which enables the virus to enter the cell by binding to the angiotensin-converting enzyme 2 receptors, lipid nanoparticle (LNP) carriers, and adjuvants [21]. Once mRNA reaches the cell, the spike protein is synthesized based on its sequence, which then stimulates IgG antibody production subsequently enabling virus neutralization [21]. Ad26.COV2-S (Jcovden) and ChAdOx1-S (Vaxzevria) are non-replicating viral vector vaccines that were applied in 9% of all COVID-19 vaccination doses in the EU [23,24,25]. Viral vector vaccines were related to lower rates of myocarditis [8]. However, they were more likely to cause thrombosis compared to other vaccines [26]. Importantly, it was shown that in the homologous vaccination schedule with mRNA vaccines, mRNA-1273 (Moderna) vaccine was associated with a higher prevalence of myocarditis than the BNT162b2 vaccine (Pfizer) [27]. The results were consistent with 4–7 excess incidents in 28 days per 100,000 vaccine recipients following BNT162b2, and 9–28 excess events per 100,000 vaccine recipients following mRNA-1273 [28]. The protein-based vaccines are PHH-1V HIPRA (Bimervax^®^, HIPRA HUMAN HEALTH S.L.U) and NVX-CoV-2373 (NOVAXOVID) which were administered in less than 1% of vaccine doses in the EU [23,29,30]. The first consists of SARS-CoV-2 spike protein from alpha and beta virus variants and the second contains a version of a protein found on the surface of SARS-CoV-2 available for the Omicron XBB.1.5 variant [23,26].

Furthermore, in terms of the most recently introduced protein-based vaccines, myocarditis and pericarditis were also observed [30]. In addition, these vaccines were related to similarly increased disproportionality as mRNA vaccines [30]. However, this was found in a retrospective study and NVX-CoV-2373 vaccines have not been used as widely as mRNA vaccines yet, thus caution in interpreting this effect is warranted and continuous observation of the potential complications related to protein-based vaccine use is needed (Table 1).

## 4. Epidemiology and Clinical Presentation of COVID-19 Vaccine-Induced Myocarditis

### 4.1. Patients with COVID-19 Vaccine-Induced Myocarditis

The increased risk of myocarditis after COVID-19 vaccination was observed in males, people younger than 30 years old, and among those who received mRNA vaccine [26]. Moreover, it was noted with relatively higher prevalence after the second dose compared to the first or third dose [9]. However, the risk of myocarditis was also increased after the first dose of the vaccine [28].

The aforementioned results, characterizing the risk factors of post-vaccination myocarditis, were consistent with other studies [27,28,31,32]. Importantly, the prevalence of myocarditis was significantly higher after vaccination in both males and females, but it was more common among males [28]. Additionally, in the studies analyzing the risk of myocarditis in children, in the age groups 5–11 years old and 11–17 years old, the risk factors of myocarditis were consistently higher in males after the second dose and after mRNA vaccines, with higher prevalence in the older subgroups [33,34]. Of note, COVID-19 infection in 2–6% of cases in the pediatric population was associated with the severe consequence of multisystem inflammatory syndrome in children (MIS-C), which in the majority of patients was associated with features of cardiac dysfunction [35,36,37]. Importantly, MIS-C associated with COVID-19 infection can be prevented with vaccination [38]. Moreover, in terms of MIS-C occurrence after vaccination, there was no increased risk observed, whereas after infection, an additional 137 (95%CI 134–140) hospital admissions from MIS-C were noted in the four to six weeks after a positive test [39].

However, there is a need to point out that, according to the risk factors of conventional myocarditis, male gender was associated with a substantially greater risk as well [40,41]. Whereas, in terms of risk stratification by age, conventional myocarditis affected a relatively older population compared to COVID-19 vaccination-induced myocarditis [41,42]. Compared to other vaccines, myocarditis after smallpox vaccination was observed mainly in males under 40 years of age, but it was a cohort study performed in the military health system, thus the 96% male dominance may be misleading [6].

### 4.2. Diagnosis of COVID-19 Vaccine-Induced Myocarditis

Diagnosis of COVID-19 vaccine-induced myocarditis should be consistent with the European Society of Cardiology’s position on general myocarditis [43]. The first-line tests concern biomarkers assessment, electrocardiogram (ECG), echocardiography, nuclear imaging, and cardiac magnetic resonance (CMR) which should be evaluated consistently with the updated Lake-Louise criteria [44]. However, CMR has to be performed if ECG, echocardiographic image, troponins, or NT-pro-BNP results are abnormal [45]. Regarding the applied CMR protocols, they should be tailored to the specific clinical situation and should typically include pre-and post-contrast T1 mapping, late enhancement imaging, standard CINE imaging, T2 edema imaging, and T2 mapping [46]. Furthermore, if a CMR image confirms myocarditis, follow-up within 3–6 months should be performed [46]. Importantly, potential viral causes of myocarditis should be excluded, i.e., COVID-19, influenza type A and B, hepatitis type B and C, Epstein–Barr-, human immunodeficiency, and cytomegalovirus infections [47].

Even though endomyocardial biopsy (EMB) is considered a golden standard in myocarditis diagnosis, due to its invasive nature it is a second-line test [38]. Moreover, it could be enhanced by molecular analysis with DNA–RNA extraction and RT-PCR amplification [43]. Importantly, EMB should be restricted to cases of post-vaccination myocarditis with acute heart failure signs, cardiogenic shock, dilated cardiomyopathy, or cardiomyopathy with constant release of inflammatory/cardiac biomarkers [46]. Additionally, EMB should be performed in patients with ventricular arrhythmias or high-degree atrioventricular block and when myocarditis is accompanied by peripheral eosinophilia [46].

### 4.3. Clinical Presentation of COVID-19 Vaccine-Induced Myocarditis

In the population with COVID-19 vaccine-induced myocarditis symptoms occurred after a median time of 3.5 days after the second vaccine dose [28]. These patients with myocarditis induced by COVID-19 vaccination presented, in the majority of cases, with chest pain, dyspnea, palpitations, general weakness, myalgia, body aches, and sub-febrile or febrile temperatures [27,46]. Acute myocarditis was often detected together with pericarditis [11]. The most common manifestation of pericardial disease is severe chest pain behind the sternum [8]. After COVID-19 vaccination, an increased risk of myopericarditis and isolated myocarditis was observed, whereas this association was not present in terms of isolated pericarditis [8]. Additionally, sometimes, vaccine-induced myocarditis was accompanied by neurological manifestations or painless thyroiditis [48,49].

Regarding laboratory features, elevated troponins were observed, with peak values within 3 days after vaccination [50]. Moreover, higher cardiac Tn-I levels predicted the severity of myocarditis, whereas high hsTnT was associated with persistent CMR lesions at 12-week follow-up among children with a median age of 15 [51,52]. However, the group of monitored children in the aforementioned study counted only 12 individuals, so these results should be confirmed in a larger cohort [52]. Other laboratory parameters that increased among vaccine-induced myocarditis patients were CRP and NT-pro-BNP or BNP [50]. The ECG changes were non-specific, mainly observed as sinus tachycardia, mild diffuse ST-segment changes, PQ segment depressions, and non-specific ST-segment changes [46]. ECG abnormalities were noted in 88.5% of patients and left ventricular ejection fraction (LVEF) lower than 50% was observed in every fifth patient [27]. However, in the Spanish multicenter registry, echocardiographic features such as left ventricular systolic dysfunction, right ventricular systolic dysfunction, and pericardial effusion were noted in 11%, 4%, and 21% of cases, respectively [53]. Furthermore, imaging characteristics of myocarditis were found in MRI among 80% of patients [54]. Among patients under 19 years of age, the main complaint was chest pain and the most common findings were ST-segment elevation and T-wave abnormalities [55].

Importantly, the main changes observed in CMR among patients with post-vaccination myocarditis were late gadolinium enhancement (LGE) of the left ventricular myocardium, typically located on the epicardial side of the lateral wall. However, low LGE volume reflected a benign course of these findings. Additionally, Lake-Louise criteria were met in 87% of cases. Moreover, abnormal T1 and T2 were found in 63% and 79% of cases, respectively [55].

Importantly, conventional acute myocarditis resolves in about 50% of cases in the first 2–4 weeks, whereas about 25% develop persistent cardiac dysfunction and 12–25% may acutely deteriorate and develop advanced dilated cardiomyopathy requiring a heart transplant [43]. Regarding post-vaccination myocarditis, symptoms resolved within 6 days in more than 90% of the patients [27,56]. In the population under 19 years of age, the mean hospital stay was almost 4 days, and in 99.67% of patients the symptoms resolved with or without treatment [57]. As some of the post-vaccination myocarditis cases can be fatal, it is needed to highlight that deaths occurred mainly among males with a mean age of 44.4 years and in 75% of cases within the first week after vaccination [58]. Furthermore, it is crucial to highlight that in the systematic review by Hulscher et al., including 14 papers that analyzed 28 autopsy cases, the cardiovascular system was solely affected in 26 of them and in the remaining two cases cardiac involvement was concomitant to systemic inflammation [58]. The most potent mechanism of death based on that study was associated with sudden arrhythmias—ventricular tachycardia or ventricular fibrillation [58].

**Figure 1:**
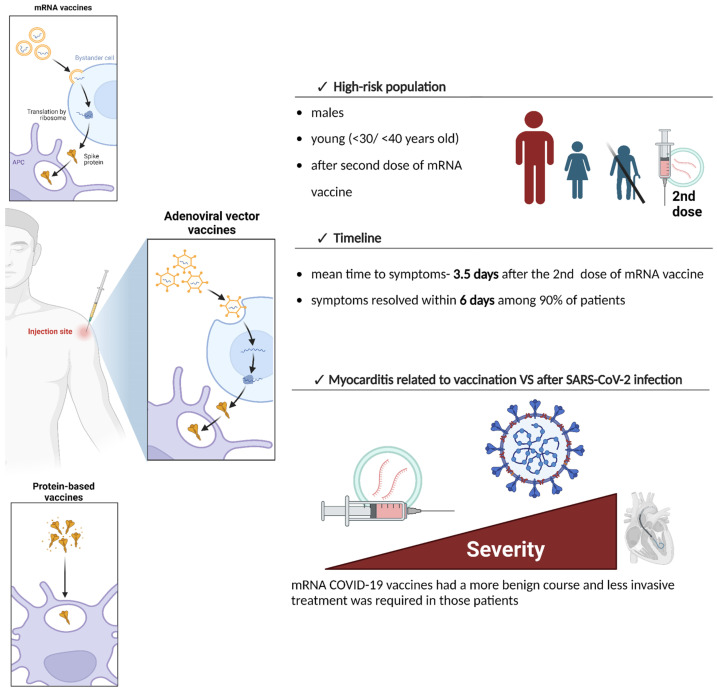
The summary of post-vaccination myocarditis-specific features. High-risk populations include males, young individuals under 30/40 years of age, and mainly those after the second dose of mRNA vaccine [9,27,30,58]. The mean time to symptoms occurrence after administration of the second dose of mRNA vaccine is 3.5 days and, in the majority of cases, symptoms resolve within 6 days [27,28,54]. Vaccine-induced myocarditis has a more benign course compared to myocarditis after SARS-CoV-2 infection [10].

Finally, myocarditis related to mRNA COVID-19 vaccines had a more benign course and less invasive treatment was required in those patients, compared to the infected patients [10]. Even though hospital outcomes were favorable, the long-term follow-up of patients who underwent myocarditis after vaccination is warranted (Figure 1).

## 5. Pathological Observations

In terms of pathological examination, myocarditis following SARS-CoV-2 infection or vaccination is not associated with any specific characteristic findings. It was shown that, similarly, in COVID-19-induced myocarditis as well as in vaccine-related inflammation, the macrophages were more commonly observed than T-cells [46,59,60].

However, the study by Schwab et al. revealed that among 25 individuals who died within 20 days after COVID-19 mRNA vaccination, the most frequent pathological finding was lymphocyte infiltration [61]. There were substantially more CD3-positive T-cells than CD20-positive B-cells found. Moreover, the majority of T-cells were CD4-positive, while sporadic CD8-positive T-cells were observed [61]. However, the analyzed cases did not match the characteristics of the mainly affected group in terms of age, sex, and the highest occurrence after a second dose [62]. In addition, another study by Kiblboeck et al. analyzed the data from 21 EMBs from patients suspected to present with COVID-19 vaccine-induced myocarditis and lymphocytic infiltration (25%), healed myocarditis (25%) was the most common, whereas myocarditis of unknown etiology was revealed as well in 25% of patients, and one cardiac sarcoidosis case was detected [63]. Eosinophils observed in the samples from EBM may suggest a mechanism of hypersensitivity to some vaccine components [64,65]. In terms of fulminant myocarditis after infection or vaccination, similar histological image was acquired in both—dominant lymphocytic myocarditis, some cases of eosinophilic myocarditis, giant cell infiltration, as well as mixed infiltration and giant cell infiltration [62,63,66,67,68,69,70,71,72].

## 6. Treatment of Post-Vaccine Myocarditis

Vaccine-induced myocarditis mostly resolves with conservative treatment and reduction of physical activity [73]. However, when needed, the optimal treatment may differ depending on symptoms. When congestive heart failure or arrhythmias are suspected, the diagnostic process and treatment should be consistent with the guidelines [14]. Among symptomatic patients, nonsteroidal anti-inflammatory medications, colchicine, and corticosteroids should be taken into consideration. This was usually effective in mRNA vaccine-caused myocarditis as well as in cases associated with vector vaccines [74,75]. With the very rare development of cardiogenic shock, it is recommended to unload the left ventricle and offer support as a bridge to recovery in cases of left ventricular dysfunction, mechanical circulatory support, and/or extracorporeal membrane oxygenation (class IIa) [14,76]. Furthermore, pediatric patients responded well to ibuprofen or other nonsteroidal anti-inflammatory drugs (NSAID), whereas in case of no improvement, additional oral corticosteroid therapy was administered with successful results [34].

## 7. Risk of Myocarditis After Infection and Vaccination

While myocarditis can arise following both SARS-CoV-2 infection and vaccination, there is a need to reveal the risk between these two scenarios. Firstly, in the meta-analysis by Volet et al., which consisted of cohorts including 55.5 million vaccinated patients and 2.5 patients with prior SARS-CoV-2, it was proven that there is a sevenfold increase in risk of myocarditis after infection, compared to vaccination [77]. Nonetheless, in the meta-analysis by Alami et al., it was shown that myo/pericarditis development within 30 days after vaccination was twice as common compared to the unvaccinated population in the absence of COVID-19 infection [78].

However, Pantone et al. performed an evaluation of more than 42 million vaccinated people over 13 years old in terms of vaccine-related myocarditis and assessed the relative risk of hospitalization or death after myocarditis due to SARS-CoV-2 infection over 28 days of follow-up [79]. The overall risk of myocarditis was greater after SARS-CoV-2 infection than vaccination [79].

Although, while concerning the stratification by age and sex it was revealed that, in men under 40 years old, myocarditis after a second dose of mRNA-1273 was more common than after SARS-CoV-2 infection [59]. Moreover, among women younger than 40 years old, the risk of myocarditis after infection and a second dose of mRNA-1273 vaccination was comparable [59]. Importantly, regarding the other analyzed vaccines, the risk of myocarditis after SARS-CoV-2 infection is greater than the risk associated with a first dose of ChAdOx1 and a first, second, or booster dose of BNT162b2 vaccine [80].

However, Buergin et al. performed a study comparing sex-specified differences in myocardial injury incidence after mRNA-1273 booster vaccination among hospital employees, which showed that an increase in high-sensitivity cardiac troponin T (hs-cTnT) was more common among women [81]. However, it is important to highlight that myocardial injury differs from myocarditis as myocarditis is an inflammatory disease of myocardium diagnosed by EMB, or in a non-invasive way by CMR [81]. Even though this study concluded that myocardial injury was more common than previously reported and occurred among 5.1% of the participants, its course was mild and none of the participants developed major adverse cardiac events (MACE) [81].

However, active surveillance would provide more valuable data than passive surveillance, whereas it is certain that overall SARS-CoV-2 infection is associated with a greater risk of myocarditis than vaccination [82].

## 8. Potential Mechanisms of Myocarditis with COVID-19 Vaccines

Even though it has been 3 years since the first cases of COVID-19 vaccine-related myocarditis were reported, the potential mechanism underlying this complication is still under debate.

While analyzing the hypothetical mechanism of vaccine-induced myocarditis, it is necessary to gain an insight into their composition, mechanism of action, and induced immunological reactions. Moreover, risk factors are supposed to be not only vaccine-dependent, but the higher prevalence of such complications in the predefined risk groups may be explained by some physiological processes. Hence, higher levels of androgens among young males, especially testosterone, may enhance the pro-inflammatory response by Th1 lymphocytes response and pro-inflammatory cytokine production such as IL-1β, TLR-4, and caspase 1 [83]. However, myocarditis was observed in the immunosuppressed children and adolescents as well, mainly after the second dose; hence, the hypothesis of an intensified systemic immunological response responsible for the myocarditis is not certain [84].

Nonetheless, it was revealed that among patients with myocarditis induced by mRNA COVID-19 vaccines, increased CD57+ natural killer (NK) cell activation was noted, as well their percentage correlated positively with the troponin T levels [85]. Genetic vulnerability was associated with the risk haplotype regarding to the killer cell immunoglobulin-like receptor (KIR) KIR2DL5B(−)/KIR2DS3(+)/KIR2DS5(−)/KIR2DS4del (+) [85]. However, NK cells destroy infected and diseased cells, thus the increase in their population may be associated with the myocarditis pathogenesis or process resolution [21].

Although the spike protein structure is very similar in all of the previously mentioned vaccines, there are suggestions that there are other components serving as antigens that could trigger the myocarditis, e.g., in the mechanism of hypersensitivity, such as adjuvants used in those vaccines, mRNA, or lipid nanoparticle (LNP) which are the mRNA carriers [30,85,86]. LNP consists of ionizable lipids which are suspected to contribute to the development of vaccine-induced myocarditis by activating Toll-like receptors (TLRs) [21,87]. Importantly, differences between mRNA-1273 and BNT162b2 and higher incidences of myocarditis after mRNA-1273 may give an insight into the probable factors contributing to its pathophysiology. Even though these vaccines have similar mechanisms of action, their carriers and mRNA structures coding the spike protein are slightly different. Moreover, the time of second dose administration varies, required storage temperature as well as the doses administered in the mRNA-1273 vaccine are higher. These factors are likely to influence the immunological processes.

One of the proposed mechanisms underlying myocarditis is the prolonged existence of mRNA that evades destruction or increased mRNA dose delivery [30]. That can cause an activation of the pro-inflammatory cascade and lead to myocarditis as part of the systemic inflammation [21]. However, it can be driven by the aforementioned hypersensitivity mechanism as well [21,85,86]. Interestingly, by immune profiling, it was found that free spike antigen was observed in patients who developed post-vaccine myocarditis, whereas it was absent in the asymptomatic patients [27]. These results are in contrast with the nationwide study in England which suggested that, due to revealed lower risk of post-vaccine myocarditis among COVID-19 survivors and lack of antibody-dependent enhancement after boosting, the spike-directed mechanisms seem to be unlikely [88].

Interestingly, it was found that within 12 months after diagnosis with myocarditis BNP levels ameliorated with the reduced third dose of BNT162b2 vaccine (0.1 mL) [89]. Moreover, low neutralizing antibody levels (<220 U/mL) after the basic scheme of vaccination were independent predictors of death or persistent BNP levels [89]. These results would bring light to the potential mechanism of drug desensitization in the reduction of myocarditis after vaccination if the hypersensitivity reaction is confirmed [90].

**Figure 2:**
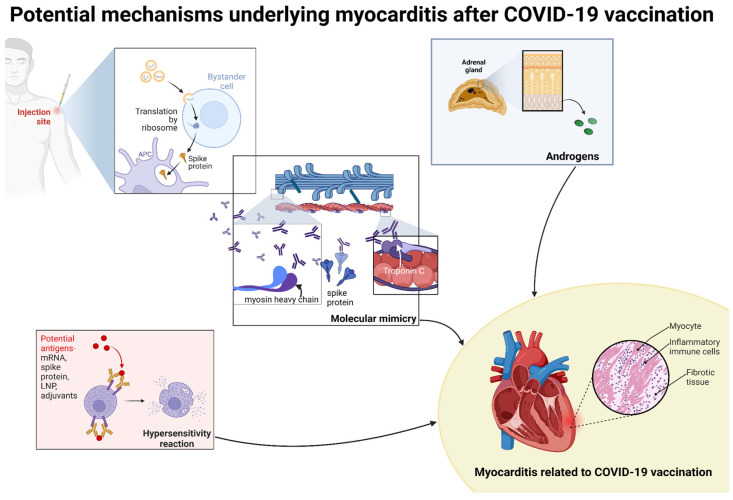
The potential mechanisms underlying myocarditis after COVID-19 vaccination are associated with higher levels of androgens, hypersensitivity reactions, and molecular mimicry [9,21,30,83,85,86]. Higher levels of androgens among young males may enhance pro-inflammatory responses by enhanced Th1 lymphocyte reaction and cytokine production, and hypersensitivity reactions may be induced by mRNA, spike protein, LNPs, or vaccine adjuvants [9,21,30,83,85,86]. Molecular mimicry is associated with a cross-reaction of antibodies against spike protein with self-antigens such as myosin heavy chains or troponin C1 [91].

Moreover, viral myocarditis was shown to be related to structural proteins of the heart and dependent on the human leukocyte antigen (HLA). The molecular mimicry between spike glycoprotein and heavy chains of myosin or troponin C1 was found to trigger antibody formation against spike glycoproteins, but at the same time against structurally similar cardiac self-antigens too (Figure 2) [91].

In conclusion, the mechanism of vaccine-induced myocarditis is still under debate. However, these immunological reactions should be considered as potential contributors and their role should be elucidated in further research.

## 9. Follow-Up among Patients with Myocarditis After COVID-19 Vaccination

Even though short-term results of patients with vaccine-induced myocarditis are favorable, the follow-up assessment is crucial to evaluate the still unknown long-term effect of these events.

Recently, the meta-analysis by Samimisedeh P et al. provided comprehensive data from follow-up of patients who underwent myocarditis after COVID-19 vaccination [92]. The assessment with CMR was performed after 3–6.3 months [92]. LGE was reported to be persistent in 76% of patients and, in nearly all cases, its extension decreased from the baseline. In six patients, persistent LGE was accompanied by myocardial edema, while in the other cases it was consistent with myocardial fibrosis [92]. Importantly, all symptoms of myocarditis resolved, ECG changes persisted in 18.7%, troponins were still elevated in 3.8% of cases, and the mean LVEF increase assessed by MRI was 2.97% [92].

Additionally, the most recent retrospective study by Talib et al., showed that among 89 patients after a mean clinical follow-up of 232 days, there were no adverse cardiac events noted [93]. However, at baseline, 47% of the analyzed group had at least one abnormality in CMR [93]. Furthermore, 12% of the patients had mild persistent symptoms of myocarditis and 48% had minimal LGE persistent [93].

Moreover, there was a comprehensive follow-up after 90 days among patients between 12–29 years of age who underwent myocarditis after COVID-19 mRNA vaccination [85]. After 90 days, 81% (320 individuals) of the patients were considered to have recovered from myocarditis. Among the considered group, at least 90% of the patients improved their echocardiogram image and their troponin concentrations were normal or returned to baseline [93]. Even though an abnormality in CMR was noted in 54% of the patients within examination performed at follow-up, findings suggesting myocarditis, such as LGE or edema, were present only in 13% of these cases [93]. Additionally, at follow-up, 68% of the patients were allowed to perform all physical activities [93].

Of note, Husby et al. compared the outcomes of myocarditis caused by SARS-CoV-2 infection, mRNA vaccination, and conventional myocarditis and the composite endpoint was defined as death or heart failure during the 90-day follow-up [94]. The lowest risk of a predefined endpoint was observed with post-vaccination myocarditis [95]. Moreover, in the group aged 12 to 39 years old, which is the high-risk group, myocarditis related to mRNA vaccination was associated with better clinical outcomes than myocarditis caused by SARS-CoV-2 infection [94].

Even though young males are affected with vaccine-induced myocarditis more often, persistent symptoms at 30-day follow-up post-diagnosis were revealed to be more common among females and older patients. Moreover, the persistence of symptoms such as chest pain, dyspnea, fatigue, and palpitations, was not predicted by the severity of the initial myocarditis image [95].

Importantly, the type of heart failure occurring during infection and after vaccination is poorly investigated and should be elucidated in further research [96]. However, during the COVID-19 pandemic, among patients with heart failure, a decline in heart failure hospitalizations was noted, whereas a pre-existing chronic heart failure or acute heart failure was associated with increased in-hospital all-cause mortality [97,98,99,100]. Furthermore, an increase in worsening heart failure and acute heart failure de novo with a further increase in in-hospital mortality, was observed [100]. However, 30-day mortality among chronic heart failure patients over 70 years of age admitted to the intensive care unit was not associated with an increased risk of mortality [101].

The impact of the COVID-19 vaccine-induced myocarditis on the long-term follow-up does not seem to be alarming. However, further follow-up is warranted as there is still not enough data from large registries to assess the impact of this myocarditis on the heart condition in the future.

## 10. Conclusions

Even though COVID-19 vaccination is associated with a risk of myocarditis, the benefits from vaccination outweigh the risk of myocarditis after SARS-CoV-2 infection. Importantly, patients with myocarditis induced by COVID-19 vaccination had a less severe course and required less invasive treatment than those who were infected.

The long-term monitoring of patients who experienced myocarditis following vaccination is warranted, despite favorable initial outcomes. Additionally, the mechanism underlying post-vaccination myocarditis should be elucidated to provide better insight into that process and potentially enable a tailored treatment for COVID-19 vaccine-induced myocarditis.

There is a need to define the predictors of post-vaccine myocarditis among high-risk groups and elucidate the risk of myocarditis after infection with the currently dominant variant. Moreover, further follow-up in terms of vaccine-induced myocarditis impact on the myocardium is warranted. Additionally, indications in terms of further vaccination for patients who underwent myocarditis after COVID-19 vaccination should be clarified. Furthermore, continuous assessments of myocarditis risk associated with various vaccines and different vaccination schemes should be conducted.

## 11. Limitations of the Study

There is a need to define the predictors of post-vaccine myocarditis among high-risk groups and elucidate the risk of myocarditis after infection with the currently dominant variant. Moreover, further follow-up and screening to avoid underreporting the impact of vaccine-induced myocarditis on the myocardium is warranted. Additionally, indications in terms of further vaccination for patients who underwent myocarditis after COVID-19 vaccination should be clarified. Furthermore, continuous assessments of myocarditis risk associated with various vaccines and different vaccination schemes should be conducted.

## Figures and Tables

**Table 1:** COVID-19 vaccine types and the risk of myocarditis.

Vaccine Name	Vaccine Type	Risk of Myocarditis
BNT162b2 (Corminaty, Pfizer)	mRNA	4–7 excess cases per 100,000 doses [27,28]
mRNA-1273 (Spikevax, Moderna)		9–28 excess cases per 100,000 doses [27,28]
Ad26.COV2-S (Jcovden)	Viral Vector	Lower risk compared to mRNA vaccines [23,24]
ChAdOx1-S (Vaxzevria)		Lower risk compared to mRNA vaccines [23,24]
PHH-1V HIPRA (Bimervax)	Protein-based	Myocarditis observed; limited data [27,28]
NVX-CoV-2373 (NOVAXOVID)		Myocarditis observed; limited data [27,28]
